# Sex Differences in Diabetic Ocular Surface Complications and Dysregulation of the OGF-OGFr Pathway

**DOI:** 10.33696/diabetes.4.052

**Published:** 2022

**Authors:** Ian S. Zagon, Joseph W. Sassani, Patricia J. McLaughlin

**Affiliations:** 1 Department of Neural and Behavioral Sciences, Penn State University College of Medicine, Hershey, Pennsylvania 17033, USA; 2 Department of Ophthalmology, Penn State University College of Medicine, Hershey, Pennsylvania 17033, USA

**Keywords:** Diabetes, Insulin, OGF, Naltrexone, Sex, Corneal surface complications

## Abstract

**Background:**

Diabetes is a chronic disorder that affects more than 500 million individuals worldwide. It is a life-long disease with complications that attack nearly all other systems within the body. Although there is a slight increase in the prevalence of diabetes in males, ocular surface complications are equally present in males and females.

**Aim:**

This review provides a discussion on preclinical studies related to the dysregulation of a biological pathway that appears to be causally related to diabetic ocular surface complications including dry eye, delayed corneal epithelial healing, and decreased corneal sensitivity. Most basic science and clinical studies focus on male sex in animal models in order to avoid confounders related to hormonal cycling. However, with approximately 10.2% of all women in the US aged 18-44 being diagnosed with diabetes and nearly 4% additional women having undiagnosed disease, it is prudent to examine the onset of these dysregulations also in females and to note any sex-related differences in the timing of onset or severity of ocular surface complications.

**Summary:**

Data from several well-controlled investigations have documented that female rats with type 1 diabetes develop ocular surface complications before male rats. In part, this finding may be due to the increase in the inhibitory peptide Opioid Growth Factor (OGF) that occurs within 2 weeks of the induction of hyperglycemia in female animals in comparison to the changes in OGF levels in male rats which occur at 4 weeks. It was noted that estrogen levels drop within weeks of induction of hyperglycemia and could serve as another marker for the onset of disease activity and/or its complications. Finally, insulin does not appear to protect against early changes in OGF levels or estrogen secretion in diabetic female rats, setting the stage for a distinction in the disease profile of diabetes between males and females. These data encourage further studies on both sexes in order to establish a complete understanding of the underlying pathologies associated with complications associated with diabetes.

## Introduction

Diabetes is a global epidemic with type 1 and type 2 diabetes affecting more than 95 million individuals in the western hemisphere [1]. An estimated 760 billion dollars are spent on diabetes and its complications annually [2]. The disease affects individuals of all ages and both genders. In the United States, approximately 10.2% of women aged 18-44 years have diabetes and another 4% have undiagnosed, pre-diabetes [3]. The CDC estimates that approximately 12.6 percent of men in the US have diagnosed diabetes and another 2.8% have undiagnosed disease [3,4]. Despite a nearly equal prevalence of type 2 diabetes between men and women, women have a greater risk for vascular complications [4] suggesting that cardiometabolic pathways and sex dimorphism in fat disposition may play a role in these gender differences. The sex inequity is magnified when ethnicity is included [5]. It is hypothesized that the breakdown of major biological pathways may be underlying the onset of diabetes and resulting complications [6,7]. Dysregulation of insulin production and/or glucose utilization are primary factors in the onset of type 1 or type 2 diabetes. Ocular surface complications of diabetes may stem from advanced glycation end products [6], as well as underlying hyperglycemia and genetics.

Data accumulated from several decades of research on streptozotocin-induced (STZ) type 1 diabetic (T1D) rat models suggest that the Opioid Growth Factor (OGF) - OGF receptor (OGFr) axis is present in the eye and is involved in growth regulation and homeostasis of corneal surface epithelium [8-12]. The OG F-OGFr axis is phylogenetically conserved and has been found in the ocular tissue of all vertebrates [13] tested to date. OGF functions to down-regulate cell division. OGF is tonically produced in an autocrine manner and requires binding to the OGFr in order to maintain a homeostatic role in cell replication. Therefore, the addition of OGF or a receptor antagonist can increase or decrease the inhibitory effects, respectively. Recently, it was determined that this regulatory pathway becomes dysregulated shortly after the onset of diabetes leading to an increased level of the inhibitory growth factor OGF in both blood and ocular tissues [14-16]. These changes in the balance of cell proliferation contribute to the onset and magnitude of ocular surface abnormalities observed in T1D. Despite the nearly equal proportion of males and females with diabetes, most preclinical studies, including our own, utilize male rodents as a model for study [e.g., 14,15], with an independent focus on female adult rats [16]. The OGF-OGFr regulatory pathway functions in normal rodents and humans, but in the diabetic model, healing of corneal epithelial defects and other ocular surface abnormalities are significantly delayed providing a reproducible model for studying the mechanism underlying delayed epithelial wound healing in diabetes. Our investigations reported that after 6-10 weeks of hyperglycemia in male rats, ocular surface complications such as low tear volume, delayed corneal epithelial healing, and altered corneal sensitivity were present [14,15]. These observations prompted experiments to identify whether female sex impacted the underlying pathways that combined the effects of repair mechanisms, diabetes and ocular surface complications were involved. Studies utilizing the genetic model of female *db/db* mice that modeled type 2 diabetes (T2D) confirmed that ocular surface complications were present in female animals and able to be modulated by the OGF-OGFr axis [17].

## Blockade of the OGF-OGFr Regulatory Pathway as Treatment

Our research involving *ex vivo* studies on rat and human corneal explants [18,19], which demonstrated that addition of naltrexone (NTX), an opioid receptor antagonist that blocks the inhibitory effects of OGF and enhanced the growth of corneal epithelial explants. Conversely, addition of the inhibitory peptide OGF resulted in little or no outgrowth. These findings prompted further investigation to determine appropriate treatment regimens using NTX. The efficacy of NTX in reversing the effects of OGF was noted after systemic [10] or topical application [11,12,14], either alone or in male T1D animals maintained on insulin [15,20] suggesting that an independent pathway not involving the etiology of diabetes (i.e., lack of or poor utilization of insulin) was involved. Many of the ocular surface defects in the male T1D rats were reversed and in some cases, non-diabetic conditions of the ocular surface were restored [15] following less than 2 weeks of NTX therapy.

## Elevated OGF Levels as a Biomarker

Our research has shifted to focus on the timing of the onset of corneal complications in T1D rats, with follow up studies exploring whether prophylactic administration of NTX beginning at the appearance of hyperglycemia can prevent or delay the onset of dry eye, deceased corneal sensitivity, and delayed corneal epithelial wound healing [14,15]. Given the relative prevalence of disease in both genders, studies on female rats were indicated [16]. The time course and magnitude of ocular surface complications and their association with the dysregulation of the OGF - OGFr signaling pathway were determined in a cohort of male and female T1D rats some of which received insulin implants in order to maintain blood glucose levels. For all groups, serum OGF levels were elevated within 4 weeks of induction of hyperglycemia, suggesting that insulin did not protect against the dysregulation of the OGF-OGFr axis. Furthermore, levels of OGF in ocular tissues including the cornea and limbus were elevated in all groups.

## Differences in Onset and Magnitude of Diabetic Ocular Surface Complications

In a side-by-side study of male and female Sprague Dawley rats rendered diabetic with intraperitoneal injections of STZ, a number of interesting comparisons were noted [21]. In addition, a subset of each sex rendered hyperglycemic was implanted with insulin minipumps to maintain blood glucose levels. Generally, body weights immediately decreased in animals with elevated glucose levels. The male rats had substantial decreases in body weight beginning on week 3 that were sustained through week 8; insulin treatment prevented the male rats from losing body weight. However, female rats showed proportionately less body weight loss; female body weights often did not differ between diabetic rats with and without insulin, despite robust changes in blood glucose. Multivariate analyses of data correlating gender, treatment, and the magnitude of the residual ocular surface defects indicated that there were significant differences between male and female Normal, T1D, and T1D-INS animals over the 8-week observation period. In general, the magnitude of complications was greater in female T1D rats than male rats. The onset of diabetic ocular surface complications appeared earlier in female T1D rats than male diabetic animals, and was associated with measurable elevations in serum OGF.

With regard to tear volume, female T1D rats displayed a deficit in tear volume within 3 weeks of being hyperglycemic, which was 2 weeks prior to effects recorded in male rats. Insulin implants at the time of initial hyperglycemia were protective against low tear production in female rats, but not in male rats, as the T1D-INS male animals had significantly reduced tear volumes beginning 6 weeks after hyperglycemia was measured. These data suggest that the dysregulation of the OGF-OGFr pathway, along with the elevated OGF blood levels may be an independent predictor of dry eye. Maintenance of normal glycemic blood levels and control of sex differences in the susceptibility to diabetic complications and/or response to insulin therapy may influence the magnitude of diabetic complications, but not prevent or reverse them.

Corneal tissue levels of OGF and OGFr were measured by confocal microscopy and optical density readings in male T1D rats at 8 weeks of hyperglycemia. Both OGF and OGFr corneal tissue levels were statistically (i.e., 2- to 3-fold) elevated above normal levels. Male rats treated with systemic NTX or topically with just one drop of NTX also had elevated OGF and OGFr tissue levels relative to their baseline or normal values. Only when NTX was administered topically twice daily for 8 weeks did OGF levels in corneal tissue match those of normal male rats. Understandably, OGF serum values were not altered by topical treatment with NTX, but were reduced following systemic NTX. OGFr levels in the serum of male diabetic rats did not respond to systemic or topical (2 drops) NTX treatment. Female diabetic rats also had substantially higher OGF and OGFr optical density measurements in ocular tissue than non-diabetic female rats. Insulin did not impact these tissue levels, but twice daily NTX drops did significantly reduce both OGF and OGFr ocular tissue levels to those observed in control female animals.

## Correlation of Ocular Surface Complications, OGF Blood Levels, and Sex Hormones

Sex hormone levels were measured in an effort to determine whether the sex differences in onset and magnitude of corneal surface complications were related to blood levels of testosterone and estrogen [20,21]. Interestingly, testosterone dropped in male animals by 70 – 80% within 2 weeks of STZ injection and remained substantially below normal (range 8-12 ng/ml) over the 8-week study period. Insulin implants did not significantly change testosterone values, nor did systemic NTX injections alter the hormone values. Estrogen levels in T1D female rats were also significantly diminished over the course of 8 weeks with a 75% drop in value occurring at 2 weeks following STZ injection. Insulin treated T1D rats had even less estrogen than T1D rats over the course of 8 weeks with values averaging 40 pg/ml relative to normal female rat values of approximately 140 pg/ml across the observational period.

OGF blood levels were not altered in either male or female rats on week 2 following STZ, but by week 3, female T1D and T1D-INS rats had elevated levels. OGF levels in males were elevated by week 5 and remained high throughout the 8-week study. Of note, OGF levels in normal and diabetic female rats were higher than their male counterparts. Beginning on week 4, female diabetic rats had blood OGF values ranging from 125 – 160 pg/ml in comparison to normal females at 50-75 pg/ml, and normal male rats with levels consistently below 25 pg/ml. The highest T1D male OGF level was approximately 60 pg/ml. Increases in enkephalin levels in diabetes have been documented in humans [22] and mice [23], but the sex differences related to the timing of the rapid increase within a few weeks of hyperglycemia is novel [14,16]. These differences may explain in part the early on set of complications in female rats. Studies to determine the mechanisms causing the increase in OGF serum levels within a few weeks of hyperglycemia are ongoing. Studies are warranted to determine how genomics and proteomics may also factor into the sex differences in the onset and magnitude of corneal surface complications [24].

## Conclusion

Multiple studies in male and female rat models of T1D confirm our hypothesis that elevated OGF and OGFr levels in serum and tissue following induction of hyperglycemia play a role in the onset and magnitude of diabetic complications involving the ocular surface, resulting in dry eye, depressed corneal surface sensitivity, and prolonged corneal epithelial wound healing. Investigations using T1D models, as well as genetic T2D female mice, confirm that treatment of this dysregulated OGF-OGFr axis using the receptor antagonist NTX effectively diminishes the complications. The timing of NTX administration is important. Early administration of NTX – as soon as hyperglycemia is detected – delays the onset and reduces the magnitude of complications, and in some cases, prevents the appearance of diabetic ocular surface complications altogether

Moreover, regulation of hyperglycemia with insulin seems to be independent of the effects of the OGF-OGFr pathway as both T1D and T1D-INS rats had elevated serum levels of OGF, and both groups responded to blockade of the regulatory pathway with NTX. As with most investigations, more questions are left unanswered. Could NTX be used prophylactically in humans with pre-diabetes? If so, would a NTX patch that slowly released the drug be feasible? Should pre-diabetic patients be screened for OGF serum levels? If OGF levels begin to increase, possibly intervention with NTX would be warranted. Limitations to the overall synopsis of data include the reliance on only female, and no male, type 2 diabetic mouse models, and no inclusion of high-fat diet diabetes animal models. Future investigations are needed to address the mechanism(s) underlying the dysregulation of the OGF-OGFr axis in diabetes, and to identify potential genetic markers in order to facilitate moving this research into the clinic so that pre-diabetes and diabetes could benefit from individualized treatment modalities.
